# Pharmacists in advanced clinical practice roles in emergency departments (PARED)

**DOI:** 10.1007/s11096-021-01275-6

**Published:** 2021-05-10

**Authors:** David Terry, Shalini Ganasan, Matthew Aiello, Chi Huynh, Veronica Wilkie, Elizabeth Hughes

**Affiliations:** 1 Aston University, Aston Triangle, Birmingham, B4 7ET United Kingdom; 2 Health Education England, London, United Kingdom; 3 School of Health and Social Care, London South Bank University, London, United Kingdom; 4 DSA Intelligence Ltd, West Bromwich, United Kingdom

**Keywords:** Advanced clinical practitioner, Emergency care, Emergency department, Pharmacist practitioner, United Kingdom

## Abstract

*Background* Following evidence published in the Pharmacists in Emergency Departments (PIED 2016) study Health Education England funded novel advanced clinical practitioner training for pharmacists (ACP-p), to support service delivery.

*Objective* To explore experiences and clinical activity of trainee ACP-p, and opinions and recommendations of both trainees and clinical supervisors.

*Setting* Five Urgent/Emergency Care Departments in London UK.

*Method* Longitudinal mixed-methods study in three phases of registered UK pharmacists appointed as trainee ACP-p. Phase 1 (May-July 2019) – early semi-structured interviews and focus group using an experiences, opinions and recommendations (EOR) framework, Phase 2 (January-December 2019) – prospective recording of trainee clinical activity, standardised using bespoke spreadsheet, Phase 3 (November-December 2019) – as Phase 1 but at conclusion of training.

*Main outcome measure* Experiences, clinical activity, opinions and recommendations of study participants.

*Results* Twelve (92 %) eligible trainee ACP-p and five supervisors were recruited. Identified themes were: trainee personality, educational components, length of programme, support/supervision, career transition, university and placement training alignment, recommendations. Success was dependent on effective support and supervision. Clinical supervisors should be allocated adequate supervision time. Trainees, their supervisors and emergency department staff should be given a clear brief. Study participants agreed that the programme could be successful. Trainee ACP-p reported that they could manage 82 % of 713 pre-selected clinical presentations. Additional training needs include: ECGs, X-rays and CT scans.

*Conclusions* Pharmacists can successfully train as ACP-p in this setting over a two-year period. This career transition needs careful management and clear structures. Training ACP-p is a useful way of enhancing skills and supporting clinical services to large numbers of patients.

## Impacts on practice

- Advanced clinical practitioner pharmacists (ACP-ps) are a potential clinical workforce and can be utilised by further training to work in the multi-disciplinary emergency department (ED) or urgent care workforce.
- ACP-ps as a form of workforce retention should be explored as part of any strategy to transition this pilot into core business across NHS Service Providers.
- ACP-ps may be an option to fill the gaps in the clinical workforce in urgent and emergency care.

## Introduction

The Pharmacists in Emergency Departments (PIED) study, published in 2017, is one of the largest studies relating to pharmacists and clinical practice [1]. After considering 18,613 patient attendances in emergency departments (ED) across 49 English hospitals the authors concluded that pharmacists have the potential to manage 36 % of all presentations. Within this context manage included diagnosis, treatment, and admission or discharge to or from hospital. PIED data was instrumental in identifying the details of further training that pharmacists would need to fulfil an advanced patient clinical management role. Health Education England (HEE) has responsibility for training over 1.3 million NHS employees in the UK [2]. HEE funded the PIED studies and commissioned new training programmes to support pharmacists in achieving their aspirations as advanced clinical practitioners [3].

In recent years a framework for training Advanced Clinical Practitioners (ACP) has been established in the UK [4]. The associated training programme allows entry of registered healthcare professionals to become ACPs. Although this is predominately nurses, pharmacists and other healthcare professionals can also enter the programme. Whilst pharmacists will bring a background in medicines optimization, all ACPs from any profession provide clinical management of patients and contribute to other pillars of ACP practice including: leadership, education and training, and research [4]. Pharmacists are eligible to train as ACPs, (denoted as ACP-p in this article) if they are registered and have suitable post-registration experience – usually two years or more. Other postgraduate qualifications are not formally required. To become recognised as an ACP-p, pharmacists must complete an approved University programme (Level 7, to at least postgraduate diploma) lasting at least one year, together with agreed clinical placements. Assessments relate to the four pillars of ACP: clinical practice, leadership, education and training, and research and include independent prescribing.

Healthcare workforce challenges in urgent and emergency care settings are recognised as a significant issue affecting NHS service delivery in the UK [5–7]. There is increased patient demand coupled with shortages in the number of doctors. European countries and elsewhere have dealt with the situation by restructuring the workforce and mobilising the clinical non-medical workforce [7–13].

From September 2016 to July 2020 Health Education England (HEE) invested in 17 pharmacists in three cohorts to train as advanced clinical practitioner pharmacists (ACP-p) within the urgent and emergency care setting. This included an 18-month full-time placement in one or more study site urgent/emergency care centres and enrolment on an agreed university-based ACP training programme.

The lack of a universally accepted definition of an advanced practice relating to pharmacists proves to be a challenge when drawing comparisons between countries. In Europe for example, literature identifying the role of advanced practice pharmacists is almost non-existent, mostly focusing on the role of the pharmacist in providing pharmaceutical care in the traditional sense in the emergency department (ED) setting and beyond [14].

### Aim of the study

To explore the experiences, opinions and recommendations of trainee ACP-p, and their clinical supervisors, who were undertaking advanced clinical practitioner training at five London teaching hospitals, and identify recommendations for further consideration of training, funding and support.

### Ethics approval

This evaluation study was approved by the Health Research Authority (HRA) on 4th February 2019 – IRAS 249,416, commissioned by HEE and sponsored by Aston University.

### Methods

Longitudinal, mixed-methods, multisite, study of trainee ACP-p and their clinical supervisors.

This study was undertaken in three phases. Phase 1 included early semi-structured one-to-one interviews (SSI) with trainee ACP-p and clinical supervisors in the current cohorts. Participant information sheets were provided and hard-copy signed consent forms obtained prior to data capture. Findings from the SSIs were used to inform a later focus group consisting of all participating trainee ACP-p. Experiences (what had actually occurred), opinions (what the participants think or feel), and recommendations (EOR methodology) formed the basis of the interview and focus group framework, and were followed sequentially according to the a priori agreed design. The study was designed and conducted in accord with COREQ principles [15]. Since it was known that there was some variability in the training experienced by the individual participants, SSIs were conducted with all to identify relevant individual details. Focus groups allowed open discussion amongst the participants and led to consensus determination of issues and recommendations. Participants were judged to be eligible if they were within six months of the start of the programme (*n* = 13, current cohorts). Interviews and focus groups were conducted by DT who was experienced in these techniques and setting, but was not previously known to the participants. Both SSIs and focus group were audio recorded, transcribed and either thematically analysed [16] using a bespoke coding framework or subjected to narrative analysis by two researchers (SG, and KCH), who produced and confirmed anonymised summary findings, reviewed by CH and DT. Early data was primarily subject to narrative analysis to identify experiences and opinions (how, why), latter data was subject to thematic analysis to support identification of recommendations (what).

Phase 2 provided the opportunity for participants to briefly record their own patient consultations using the method described by Hughes et al. [1]. Data recorded was: date, location, patient gender, age, presenting complaint, clinical grouping, clinical skills required to manage patient, trained ability to manage patient (yes/no), further clinical skills needed if any, are these skills included in your training programme and comments. In this manner patient presentations, skills required to manage the patient and any personal skill gaps were identified. Records were completed using a bespoke online form designed in MS Excel 2013. Training was provided to the participants on how to complete these records. The study team monitored the records and had opportunity to provide further instruction on how the form was to be used if needed.

Phase 3 mirrored the methods used in Phase 1 but was conducted at the end of training programme, and focused on later stages of the programme and final recommendations identified in analysis of focus group and semi-structured interviews.

Data was stored on AES-256 protected electronic files approved for the purpose by Aston University.

## Results

12 trainee ACP-p participated, with one trainee ACP-p declining.

Prior to joining the programme the trainees, all registered and experienced pharmacists, came from a variety of backgrounds, including:

- Nine hospital pharmacists.
- One interface pharmacist (between primary and secondary care).
- One pharmacist from primary/community care.
- One pharmacist from primary/secondary care.

Post-registration experience ranged from 3 to 8 years.

Four medical doctors and one advanced nurse practitioner participated as clinical supervisors across the five study sites. Each supervised more than one trainee ACP-p.

### Experiences and opinions of trainee ACP-p

#### Trainee resilience

The majority of the ACP-p trainees said that they needed to be mentally strong and resilient to undertake the programme and to complete what is a ‘drastic’ change in their professional working lives. They expressed the view that it’s a difficult transition, and future trainees need to consider this carefully and be prepared for the challenges ahead.

I am the type of person to get on with things, in my opinion this helped the challenging transition period, but I appreciate that not everyone is like that you know and so if you’re not like that it would’ve been a struggle. P5, Hospital Pharmacist.

### Programme delivery and alignment

The ACP-p trainees felt they were disadvantaged by training site contracts lasting 18-months when the University programme lasted two years. They recommend that these periods of study should be aligned.

The job was advertised for 18-months whilst the postgraduate ACP programme is for 2 years. There seems to be a disconnect between Trust competency requirement and university requirements. These should be aligned. P4, Hospital pharmacist.

### Educational programme

The ACP-p trainees found the initial induction organised by Health Education England (HEE) to be useful and where hospitals/Trusts organised an additional induction to supplement the first induction, it helped them transition into the role.

We had a 2-week introduction kind of timetable I believe from Health Education England when we started, with all the other ACP – p trainees from the different Trusts. Our ACP lead at the Trust also designed an induction timetable for us in the first month … If this wasn’t in place, I think it would have been very difficult to make that transition. P9, Hospital pharmacist.

The ACP-p trainees commented that achieving competence from a standing start in 18-months is a significant challenge for many, and a longer training period may be more realistic.

I think HEE needs to know that it`s not an 18-month programme it’s a 3-year programme. P4, Hospital Pharmacist.

Trainees benefited from the ACP course at the university especially in the early stages of training when they lacked clinical assessment skills.

Our first module was physical assessment and I think it was actually quite a good way of introduction into the programme. P10, Primary and Secondary Care Interface pharmacist.

It was noted that the trainees found the Trust-based training for junior doctors useful in terms of emergency care teaching.

The FY2 teaching is delivered by the A&E consultants and every week a different topic is discussed. It is a very well structured and focused sort of training. P9, Hospital Pharmacist.

### Support

Some training sites had little previous exposure to pharmacists within their Emergency Departments, and where this is the case the role of pharmacists may be poorly understood and sometimes challenged. Sites which were well prepared for the introduction of pharmacist trainees generally provided a more comfortable learning experience for the pharmacists and helped with integration into the team.

Some sites were described as welcoming and supportive.

I think our set up is good in that before we arrived people knew who we were. I think you really need all doctors in the department and the team to know who you are because it helps you integrate. P11, Hospital Pharmacist.

Some sites were described as ‘hostile’.

Lack of acceptance and hostility within the ED environment made me feel unsupported and unwelcome. P12, Primary Care and Hospital Pharmacist.

### Supervision

Supervision varied between the sites. Trainees benefiting from effective and accessible clinical supervision reported a good experience.

My clinical practice is supervised on an ad hoc basis by a consultant, no formal supervisor appointment to the position for about 12 months. As a result, I am planning to move on to a role where I will be able to develop my skills and be supervised by a nominated supervisor. P3, Hospital Pharmacist.

Excellent training base as holistic and well-rounded training with varied practitioners available. Several practitioners have also undertaken the ACP course and understand the learning needs from academic/university and professional/practice-based perspective. This resulted in a positive experience during my training period. P1, Hospital Pharmacist.

### Experiences and opinions of the clinical supervisors

#### Trainee response

The clinical supervisors’ findings mirrored the pharmacists’ opinions in terms of trainee response.

I think some of them have really picked things up very quickly depending on personality types and I think others have not picked it up as quickly and I would say that it’s similar to what we have seen in nurses and doctors. P8, Consultant in Emergency Department.

### A challenging experience

Clinical supervisors confirmed this was a challenging programme and a first of its kind.

The programme is really exploring uncharted territory and is more embryonic than originally described and expected. Clinical pharmacists have very limited experience working in Urgent Care settings. P15, Consultant in Emergency Department.

In addition, differences in pay banding and salary introduced tensions among non-medical colleagues.

Students (ACP-p trainees) are training and paid at 8a – this exceeds expectations of other groups (e.g. nurses) with consequential problems. P15, Consultant in Emergency Department.

### Programme delivery and alignment

The mismatch between contract of employment lasting 18-months and university academic course running over 2 years proved to be problematic.

I could not understand why it was 18 months, as far as I am concerned it is 2 years. P17, Advanced Nurse Practitioner.

### Educational element

It was identified that ACP-p trainees may need a longer period to consolidate their knowledge in the emergency department setting.

Pharmacists need a longer period to consolidate their knowledge than our nurse ACPs who have spent longer seeing patients in the emergency department. P8, Consultant in Emergency Medicine.

The background training required includes modules such as anatomy.

The ACP pharmacist trainees’ actual clinical background was inadequate e.g. anatomy … P15, Consultant in Emergency Medicine.

In the UK pharmacists can undertake further post-registration training to become fully independent prescribers. It was identified that completion of prescribing training prior to the study may be useful.

In retrospect, not having prescribing first made it too much to do in 18 months. P14, Consultant in Emergency Medicine.

The knowledge required at the start of the programme is best acquired in another setting instead of in the emergency department.

I think it would have been better if they had some of the academic course before coming to us. P18, Urgent and Emergency Care Consultant / GP.

### Support

The clinical supervisors found their own initial induction to be of limited benefit.

HEE provided an initial short training–2 weeks–covering some basics, which was useful but of limited benefit. P15, Consultant in Emergency Department.

The requirement to develop ACP-p in merely 2-years was a challenge.

When they`ve come from a very different background to support effectively, shoehorn all that training, all the theoretical knowledge that they need plus the practical knowledge into just two years, I think it is quite unrealistic expectation. P8, Consultant in Emergency Medicine.

The extent of support required by the trainees should not be underestimated.

You need to be aware that these trainees will need a lot more support in the early stages–which to be fair would be pretty obvious. I certainly didn’t feel that it was any more onerous for the senior clinical team to support them as opposed to other SHOs. Because they were very enthusiastic/seeking out training opportunities, helped them in that regard. P16, Emergency Medicine Consultant.

### Supervision and governance

The supervision and competence element created unrest amongst the clinical supervisors.

The clinical supervisors are being asked to sign the trainees off as independent at the end of the first year, by University, when it was never the intention that they would be independent after only one year. P15, Consultant in Emergency Medicine.

Lack of protected supervision time and extent of supervision required by ACP-p trainees was unsatisfactory to clinical supervisors.

These ACP trainees really need a lot of support and a lot of supervision by a consultant and we raised the fact that we don’t have that capacity. P8, Consultant in Emergency Medicine.

Governance was initiated by the Trust with minimal or lack of input from HEE or the university.

Governance of the programme locally has been provided by the ACP Governance group–chaired by the Medical Director–who meet each month to consider progress, approach and actions. This is a bespoke group for this training programme. P15, Consultant in Emergency Medicine.

### Project future and recommendations

Despite its shortcomings, this project could be successful with support from all stakeholders involved, and initial training to support ACP-p transition in their new roles.

The programme can work with commitment from all concerned (Trust, senior management, staff), clarity from the organisations over the programme arrangements, and significant effort from the pharmacists themselves to engage as clinical students in an unfamiliar environment. P15, Consultant in Emergency Department.

The brief from HEE should be more structured and Trusts need to keep an open mind about the pilot.

If HEE is looking to promote this programme, as there is value, HEE needs to be more specific of what types of roles they want to fill and expectations of what period of time, these practitioners can be independent mid grades … P16, Emergency Medicine Consultant.

There is definitely a role for ACP-p.

I would welcome the next cohort without doubt. When nurses started as ACP, it wasn’t accepted very well. The ACP-p trainees need to see why they want to do it, what their expectation is, have an understanding of what they are going to be doing so it gives them an idea to see if they want to take on the challenge and culture shock. P17, Advanced Nurse Practitioner.

### Clinical activity data

Nine trainee ACP-p recorded details of 713 patients that they saw during the study in the period January to December 2019 from four study sites.

Table 1 below shows the number of patients they saw in each clinical grouping as defined by Hughes et al. [1], and the number that they considered they could independently manage within their own skill set at the time. Patients were selected according to the trainees’ skills and training needs at the time of presentation. Trainees were supervised as part of the multidisciplinary team. Competence to manage was not formally assessed by another trained practitioner at an individual patient level.

**Table 1:** Numbers of patients recorded in clinical groups seen by trainee ACP-p and numbers that they considered they were competent to independently manage–all study sites

Clinical Grouping	Independently manage = Yes	Total	%Yes
Medical-general	154	174	89 %
Respiratory	85	106	80 %
Cardiology	55	74	74 %
Gastrology	59	66	89 %
Other	48	58	83 %
Neurology	44	55	80 %
Urology	27	33	82 %
Orthopaedics	20	28	71 %
Surgical-general	21	26	81 %
Ear-Nose-Throat	20	24	83 %
Trauma	20	24	83 %
Obstetrics and Gynaecology	11	14	79 %
Pain	12	14	86 %
Renal	7	12	58 %
Liver	3	3	100 %
Paediatrics	1	2	50 %
Totals	587	713	82 %

Table 2 shows the numbers and proportions of patients recorded by trainee ACP-p that they considered they were competent to manage by site.

**Table 2:** Numbers and proportions of patients seen by trainee ACP-p at four study sites

Site	Manage = Yes	Total
1	207 (72 %)	287
2	77 (76 %)	101
3	236 (92 %)	256
4	67 (97 %)	69

Trainees were asked to identify their own specific training needs to manage patients they saw, at the time of the consultation.

Training needs were recorded for 179 (25 %) patients. Of these, the trainees identified two or more training needs for 47 individual patients.

Table 3 shows the category, sub-category and frequency of training needs recorded by the ACP-p trainees.

**Table 3:** Category, sub-category and frequency of training needs recorded by trainee ACP-p

Category	Sub-category	Frequency
ECG		49
X-ray	All	42
	Chest n=27	
Computed tomography	All	33
	Head n=15	
	Kidney ureter bladder n=5	
	Chest n=4	
Prescribing	All	10
	Fluids n=3	
Ultrasound	All	8
	Abdominal n=3	
Arterial blood gases		5
Fundoscopy		5
Rectal examination		5
Venous blood gases		4
Wound care		4
Cardiac monitoring		3
Mental health		3
Spirometry		3
Heart murmurs		2
Injuries (general)		2
MSK		2
Nystagmus		2
Pain management		2
Spine assessment		2
Stroke		2
Ultrasound		2
Wrist injuries		2
Other		40
Total		232

Twenty recommendations were identified and are summarised in Table 4 below.

**Table 4:** Recommendations in relation to ACP-p training by category

1	Sites	Provide effective support and supervision
2	Sites	To be made aware of the additional training burden on staff
3	Sites	Ensure ED staff are aware of programme and trainee needs and role.
4	Sites	Beneficial if the ED department has had prior exposure to clinical pharmacists
5	Sites	Beneficial if the ED department has commitment to long term employment of ACP-p
6	Trainees	Be prepared for the challenges of transitioning to a new role
7	Trainees	Be prepared that this is a training role, requiring significant personal study time in addition to placement work time.
8	Supervision/ supervisors	Provide effective, protected and accessible clinical supervision
9	Supervision / supervisors	Supervisors to receive adequate induction and attend University training day.
10	Supervision/ supervisors	Support of ACP-p trainees should not be underestimated, who may require more supervisor time that trainees from other professions.
11	Supervision/ supervisors	Trainees may need more than one supervisor.
12	Training programme	Align University and placement periods
13	Training programme	Provide organisational and local inductions
14	Training programme	Minimum of two years
15	Training programme	Placement training with junior doctors
16	Training programme	Provide pre-programme training (e.g. anatomy etc. in a bridging module) and experience (in another clinical environment)
17	Training programme	Where possible trainees to complete independent prescribing prior to ACP-p training
18	Training programme	Include early training in ECGs, Chest x-rays and CT scans
19	Health education England	Provide a clear description of the programme to all interested parties, including written Job Descriptions and support arrangements
20	Health education England	Recruit sites, supervisors and trainees carefully. Sites require a supportive team, supervisors need adequate time, and trainees must be willing to study outside of work time.

## Discussion

### Sites

Experiences and opinions of participants were not always aligned. It is evident that Trusts providing effective support and supervision created a more welcoming environment for the pharmacists.

There is a paucity of evidence identifying exact number of training hours that translate into core competencies [17]. It is recommended that these be identified and communicated consistently to all pilot sites for similar projects in the future. This would enable trainees to be aware of what is expected of them, types of patients they will see and which areas of urgent or emergency care they will work in.

This study highlights the importance of appreciating that immediate reduction in workload may not be seen. Significant support is required in the interim period [18]. Clinical supervisors noted the training burden on Emergency Department staff, and any extra training arrangements for ACP-p trainees need to be carefully considered at each site.

Clarity of new roles as they are introduced is essential and this reflects findings in many workforce development programmes [19]. Evidently, this promotes ease of integration for the trainees into a fast paced and rapidly changing environment for new members of the team [10]. Trainees reported negative impact when staff in the ED were not aware of why they were there and the extent of their new role. This finding matches evidence documented in literature on integration of pharmacists into the urgent care setting [20].

Liberati identified that integration is impeded by the need to defend ‘…discipline specific professional identities, specialised knowledge and pre-existent practices’ [21]. This is supported by literature addressing resistance to collaboration if one’s role is perceived as being threatened [22]. At some training sites it was noted that there is a tension between existing non-medical staff and the ACP-p trainees, perhaps prompted by salary differentials and roles.

The study participants noted the benefit of placing students at training sites who have a genuine interest in the future employment of ACP-p, as opposed to sites simply willing to provide the training without any future expectations.

### Trainees

The journey - transitioning into this autonomous role - was in parts challenging for the trainees.

Trainees need to be resilient and proactive [23] not so much in dealing with the work of the Emergency Department, but rather about the transition in roles that they are likely to experience. Shop-floor clinical teaching is not the norm for pharmacists compared to medical doctors [24]. Currently pharmacists lack clinical assessment and diagnosis training at undergraduate level compared to their medical counterparts [24, 25].

It was identified that ACP-p trainees should make full use of the wide range of teaching materials available to them and put in considerable time both within the learning environment and away from it to enhance their learning. Literature highlights the disadvantaged positions that pharmacists are in compared to nurses when embarking on advanced roles [26]. The lack of patient contact, anatomical knowledge and diagnostic skills were identified as potential barriers to encroachment of traditional roles of nurses and doctors [27, 28].

Records of clinical activity confirm the ACP-p trainees saw a wide range of patients, with 82 % considered by the trainees themselves to be within their competence to manage. Unlike the PIED study [1] where pharmacists were asked to assess a representative sample of all ED patient presentations, in this present study ACP-p trainees recorded activity with patients they were directed to, or chose to manage. The ability to manage these targeted patients was self-assessed by the trainees themselves. It is therefore not surprising that their ability to manage large numbers of patients is confirmed in this study.

### Supervisors/supervision

Good and effective supervision was found to be helpful in alleviating the anxiety associated with the transition to a new role [29]. Given the novelty of this project, supervisors were mostly physicians. Barton identifies the challenges with one professional body mentoring another as highlighted by some ACP-p trainees [30]. A description of the trainees’ educational backgrounds to inform prospective training sites of their clinical training would be beneficial.

Clinical supervisors should attend the University programme training day and be given sufficient information to support realistic expectations of ACP-p trainees. More than one clinical supervisor may be necessary in order to cover absences especially where there are a group of trainees at the site. Literature explains that the supervision arrangement whether formal or informal influences the success of the mentorship [31].

### Training programme

ACP-p trainees welcome a structured and agreed training pathway with suitable inductions. Aligning academic courses with training placements is essential. It is recommended that a bridging module be introduced prior to the trainees embarking on the ACP course and that the duration of the ACP and bridging course be longer than 2 years. It may be advantageous for independent prescribing training to be completed before or early in the training programme. In the near future the General Pharmaceutical Council expects pharmacists to be independent prescribers at the time of professional registration.

Some exposure to the environment and associated skills required prior to undertaking a training role will be advantageous.

Further training relating to ECG, X-ray and CT scans are identified as priorities.

### Health education England (funding body)

Both ACP-p trainees and their clinical supervisors observed that HEE provided very limited guidance concerning the training programme. HEE should provide a clear description of the programme to all stakeholders, highlighting required actions by NHS training sites.

Study training sites should be chosen carefully according to the support they can offer trainees. It may be beneficial to choose sites that have some experience of working with pharmacists that are able to provide a structured training programme integrated with other professional trainees.

It is the recommendation of this study that educational and clinical supervisors should be carefully chosen perhaps following interviews, provided with a written job description and supported from the Trust and HEE alike. Supervisors should be advised of the strengths and weaknesses of trainees from a pharmacy background, and given sufficient time to provide appropriate support.

The limitations of this study include metrics were not gathered to estimate development of skills or contribution to workload. In addition, there were limited published literature on ACP-p [32]. Most advanced practice literature involves nurses. There exist fundamental differences in the training, career progression and governance structure for each of the professions respectively [23]. Another limitation was that the participants were at different stages on the programme at any one time. Feedback may have been skewed to how they felt at the point of data collection. All participants worked in slightly different environments and scope of practice varied according to Trust requirements and governance structures. Trainees self-assessed their ability to manage patients, which was not independently verified by a trained practitioner.

Overall, participants consider the training programme evaluated in this study to be adequate to support pharmacists to become ACP-p, but can be much improved. All stakeholders should consider and agree actions in response to the recommendations, to enable all concerned to be prepared for this challenging transition in career.

Further work is required to determine if the identified recommendations lead to practical and deliverable training for pharmacists transitioning to ACP-p.

## Conclusions

Recommendations have been identified that will improve ACP-p future training programmes. The transition from traditional pharmacy roles to those of an ACP-p is challenging, but achievable. Programme providers should ensure that sites are suitably supportive, supervisors are aware of the challenges and given sufficient time to fulfil their training role, and trainees need to commit to intensive training beyond what can be achieved in the normal working day. In-practice and university training need to be aligned.

Developing ACP-ps has a future and should be considered when developing workforce initiatives. Training ACP-p is a useful way of enhancing skills, providing a new career pathway and encouraging staff retention as outlined in the NHS Interim Plan [33].
